# Draft Genome Sequence of Staphylococcus epidermidis UMB7543, Isolated from a Female Patient with Recurrent Urinary Tract Infections

**DOI:** 10.1128/mra.00962-22

**Published:** 2022-11-10

**Authors:** Sandra Jablonska, Alan J. Wolfe, Catherine Putonti

**Affiliations:** a Department of Biology, Loyola University Chicago, Chicago, Illinois, USA; b Bioinformatics Program, Loyola University Chicago, Chicago, Illinois, USA; c Department of Microbiology and Immunology, Loyola University Chicago, Maywood, Illinois, USA; University of Rochester School of Medicine and Dentistry

## Abstract

Staphylococcus epidermidis is a Gram-positive bacterium that is part of the normal human flora, found in multiple anatomical sites. Here, we present the 2.6-Mbp draft genome sequence of S. epidermidis UMB7543, isolated from a catheterized urine sample from a female patient with a documented diagnosis of recurrent urinary tract infection.

## ANNOUNCEMENT

Staphylococcus epidermidis is the most common source of infections from medical devices, including indwelling catheters (1, 2). These infections can be chronic and persistent due to the production of biofilms, which often confer antibiotic resistance (3, 4). Much more frequently, S. epidermidis is a benign member of the human epithelia. While it predominantly colonizes the skin, it can also be found in other organs, e.g., the urinary and gastrointestinal tracts. Here, we present the draft genome sequence of S. epidermidis UMB7543, isolated from a catheterized urine sample from a postmenopausal female patient with a recurrent urinary tract infection (rUTI). Although S. epidermidis has previously been associated with UTIs in children (5), this strain is not believed to be the cause of UTI symptoms for this individual.

S. epidermidis UMB7543 was isolated using the expanded quantitative urine culture (EQUC) method (6), as part of a prior institutional review board (IRB)-approved study (University of California, San Diego; IRB no. 170077AW). The genus and species for this isolate were determined by matrix-assisted laser desorption ionization–time of flight (MALDI-TOF) mass spectrometry following protocols detailed previously (7). The isolate was stored at −80°C until sequencing. The freezer stock was streaked onto a Columbia nalidixic acid (CNA) agar plate and incubated at 35°C with 5% CO_2_ for 24 h. Tryptone soy liquid medium was inoculated with a single colony from the plate and incubated overnight at 37°C. DNA extraction was done using the Qiagen DNeasy blood and tissue kit; the manufacturer’s Gram-positive protocol was followed with an altered prelysis step (previously described) (8). The purified DNA was quantified using a Qubit fluorometer. DNA sequencing was performed by SeqCenter (Pittsburgh, PA). There, sample libraries were prepared using the Illumina DNA prep kit and IDT 10-bp unique dual indices (UDI) and sequenced on the Illumina NextSeq 2000 platform. Sequencing yielded 1,167,425 pairs of 150-bp reads. Unless otherwise noted, default parameters were used for all software tools. The reads were trimmed using BBDuk v37.36, which is part of the BBTools suite (https://jgi.doe.gov/data-and-tools/software-tools/bbtools/). The reads were assembled using SPAdes v3.15.4 with the “only-assembler” option for k values of 55, 77, and 99 (9). The genome coverage was calculated using BBMap v38.47, also part of the BBTools suite. NCBI’s Prokaryotic Genome Annotation Pipeline (PGAP) v6.2 was used to annotate the publicly available genome assembly (10).

The draft genome sequence is 2,527,127 bp long, assembled into 57 contigs, with an *N*_50_ value of 98,169 bp and 66.75× coverage. The GC content of the assembled genome is 32.1%, which is consistent with that of other publicly available S. epidermidis genomes. PGAP annotations included 2,296 genes total, 2,296 encoding proteins, and 56 tRNAs. No antibiotic resistance genes were found within the genome assembly using ResFinder v4.1 (11). PHASTER analysis of the draft genome found one incomplete and one intact prophage (12). When queried against the nonredundant/nucleotide (nr/nt) database via MegaBLAST, the intact prophage sequence, 55.1 kbp, most closely resembled metagenome assemblies of phages. Despite high percent identities (>90%), these hits often had low query coverage (10 to 63%). When queried against the refseq_genome database of viruses using MegaBLAST, the greatest sequence similarity was to the characterized Staphylococcus phage vB_SepiS-philPLA5 (GenBank accession no. NC_018281.1) (query coverage, 15%; identity, 93.62%).

### Data availability.

This whole-genome shotgun project has been deposited at GenBank under the accession no. JANRMH000000000. The version described in this paper is the first version, JANRMH010000000. The raw sequencing reads have been deposited in the SRA under the accession no. SRR21102740.
